# Thermosensitive hydrogel with programmed dual-octenidine release combating biofilm for the treatment of apical periodontitis

**DOI:** 10.1093/rb/rbae031

**Published:** 2024-03-26

**Authors:** Yu Cheng, Yini Huangfu, Tingyuan Zhao, Linxian Wang, Jing Yang, Jie Liu, Zujian Feng, Kehua Que

**Affiliations:** Department of Endodontics, School of Stomatology, Hospital of Stomatology, Tianjin Medical University, Tianjin 300070, China; Tianjin Key Laboratory of Biomaterial Research, Institute of Biomedical Engineering, Chinese Academy of Medical Sciences and Peking Union Medical College, Tianjin 300192, China; Department of Endodontics, School of Stomatology, Hospital of Stomatology, Tianjin Medical University, Tianjin 300070, China; Department of Endodontics, School of Stomatology, Hospital of Stomatology, Tianjin Medical University, Tianjin 300070, China; Department of Oral Implantology, Tianjin Stomatological Hospital, Tianjin 300041, China; Department of Endodontics, School of Stomatology, Hospital of Stomatology, Tianjin Medical University, Tianjin 300070, China; Tianjin Key Laboratory of Biomaterial Research, Institute of Biomedical Engineering, Chinese Academy of Medical Sciences and Peking Union Medical College, Tianjin 300192, China; Department of Endodontics, School of Stomatology, Hospital of Stomatology, Tianjin Medical University, Tianjin 300070, China

**Keywords:** thermosensitive hydrogel, programmed drug release, antibiofilm, intracanal medicament, apical periodontitis

## Abstract

The utilization of intracanal medicaments is an indispensable procedure in root-canal treatment. However, the conventional intracanal medicaments still need improvement regarding antimicrobial efficacy and ease of clinical operation. To address the above issues, OCT/PECT@OCT + ALK composite hydrogel characterized by programming sequential release of dual antimicrobial agents has been proposed. Thanks to the self-assemble ability of amphiphilic copolymer poly(ε-caprolactone-co-1,4,8-trioxa [4.6]spiro-9-undecanone)-poly(ethylene glycol)-poly(ε-caprolactone-co-1,4,8-trioxa[4.6]spiro-9-undecanone) (PECT), dual hydrophilic and hydrophobic antimicrobial agents could be easily encapsulated in the hydrogel system and tailored for sequential drug release for a better antibiofilm effect. The hydrophilic octenidine (Octenidine dihydrochloride, OCT-HCl) is encapsulated in the hydrophilic part of hydrogel for instantaneous elevating the drug concentration through bursting release, and the hydrophobic octenidine (Octenidine, OCT) is further loaded into the PECT nanoparticles to achieve a slower and sustained-release profile. Additionally, calcium hydroxide (Ca(OH)_2_) was incorporated into the system and evenly dispersed among PECT nanoparticles to create an alkaline (ALK) environment, synergistically enhancing the antibiofilm effect with higher efficiency and prolonged duration. The antibiofilm effect has been demonstrated in root-canal models and apical periodontitis rats, exhibiting superior performance compared to clinically used Ca(OH)_2_ paste. This study demonstrates that OCT/PECT@OCT + ALK composite thermosensitive hydrogel is a potential intracanal medicament with excellent antibiofilm effect and clinical operability.

## Graphical abstract

**Figure:**
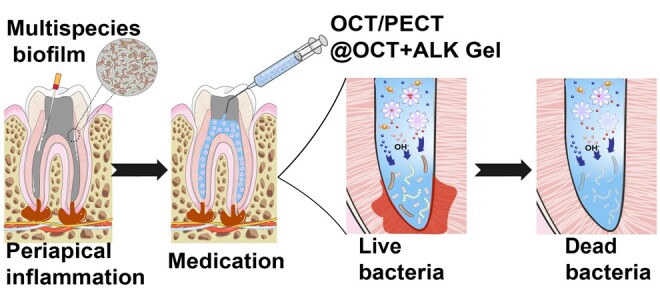

## Introduction

Root-canal treatment (RCT) is currently the first choice for the therapy of pulp infection and periapical inflammation. The primary objective of RCT is to thoroughly eliminate microorganisms within the root canal while preventing reinfection through sufficient mechanical preparation and disinfection [1]. The widely utilized root-canal disinfectants in clinics include calcium hydroxide (Ca(OH)_2_) paste, chlorhexidine (CHX) hydrogel and triple antibiotic paste [2, 3]. However, Ca(OH)_2_ pastes exhibit insufficient antibacterial efficacy against the complex biofilm [4]. Due to the dosage form of current clinical Ca(OH)_2_ pastes, complete removal of Ca(OH)_2_ adhered onto the root-canal wall is often difficult, and subsequent further root-canal filling becomes inconvenient [5]. Furthermore, studies have shown that the abuse of antibiotics such as ciprofloxacin and metronidazole in root canals can lead to drug resistance of bacteria, such as *Enterococcus faecalis* and *Candida albicans* [6]. Ultimately, anti-infection efforts are impeded and the efficacy of drugs is diminished. Therefore, it is imperative for further research in root-canal disinfection to focus on exploring intracanal medicaments that exhibit superior antibacterial effects and provide greater convenience during clinical procedures.

Apical periodontitis can be classified into acute or chronic types. Acute infection is usually caused by a highly virulent bacterial community, leading to a high concentration of bacteria in the root canal and invasion of periapical tissues [7]. Conversely, chronic infection typically involves low-virulence bacteria that remain hidden in the root canal and cause long-term irritation of periapical tissues, resulting in chronic inflammation [8]. Studies have indicated that persistent bacterial infection is the most significant factor contributing to the failure of RCT [9]. The microbiota responsible for such infections is highly diverse. Several studies have demonstrated a significantly higher ratio of gram-positive facultative anaerobic bacteria, including *E. faecalis*, *Actinomyces viscous* (*A. viscous*), and *Streptococcus mutans* (*S. mutans*) in root-canal infections [10, 11]. These bacteria primarily exist in the form of biofilm, which adhere onto the inner dentine surface, lateral/accessory canals and isthmus of the root. It may also reside on the outer root [12]. Therefore, combined with the above-mentioned factors, the ideal intracanal medicaments are supposed to possess sequential characteristics that enable them to control acute infection in the root canal by using burst-release drugs during the early stages of application. Simultaneously, sustained antimicrobial effects are maintained through slow release to prevent the occurrence of reinfection over an extended period.

Hydrogel is a three-dimensional porous material composed of physically or chemically crosslinked polymer chains, with excellent hydrophilicity and biocompatibility. It can be loaded with small-molecule compounds to achieve sustained drug release and is extensively applied in various fields, such as wound dressing, tissue engineering and regenerative medicine [13, 14]. The gradual advancement of hydrogel drug-delivery mode in the dental field also presents a novel direction for intracanal medicaments [15]. The thermosensitive hydrogel, as a specialized type of hydrogel, remains in liquid state at room temperature and rapidly transforms into semi-solid or solid hydrogel upon injection at body temperature, demonstrating remarkable adaptability to the intricate structure of the root canal. It can also be loaded with various drugs based on clinical requirements to attain antibacterial efficacy [16]. Poly(ε-caprolactone-co-1,4,8-trioxa [4.6]spiro-9-undecanone)-poly(ethylene glycol)-poly(ε-caprolactone-co-1,4,8-trioxa[4.6]spiro-9-undecanone) (PECT) is a representative thermosensitive hydrogel, which is a modified poly(ethylene glycol) (PEG)/polycaprolactone (PCL) polymer by pendant cyclic ether with many unique characteristics, including injectability, biocompatibility, high drug-loading content and controlled drug delivery [17]. PECT hydrogel has been applied in various fields such as peritumoural injection of paclitaxel (PTX) and doxycycline or peri-implantitis treatment loaded with ibuprofen and basic fibroblast growth factor, which is a promising drug-delivering platform [18, 19]. Owing to the amphiphilic property of PECT polymer, it can self-assemble into nanoparticles (NPs) with a regular hydrophilic shell around a hydrophobic core, enabling the simultaneous loading of hydrophobic and hydrophilic drugs in the hydrogel system to exert diverse functionalities [20]. Moreover, the drug-release profile can be easily tailored by adjusting the hydrogel components. Therefore, we hypothesized that PECT can be a promising candidate for programming the delivery of antimicrobial agent to control acute and chronic infection in apical periodontitis.

In the present study, PECT thermosensitive NP hydrogel with programming sequential dual drug-release profile was rationally prepared for apical periodontitis treatment. OCT, a broad-spectrum antimicrobial used in clinics [21], was selected owing to its inherent hydrophilic and hydrophobic form according to whether it is demineralized. The hydrophilic OCT was encapsulated in the hydrophilic part of the hydrogel system for burst release to control the acute infection at the early stage. The hydrophobic OCT was loaded into to PECT NPs to obtain a long-term sustained drug-release profile for preventing the occurrence of reinfection over an extended period. To further enhance the antimicrobial effect of this hydrogel system, low-dosage Ca(OH)_2_ was introduced to create a long-term higher ALK environment while attempting to solve the operational difficulties associated with it in clinical settings. Applying it as an intracanal medicament to achieve both short-term and long-term antibiofilm effects can provide a new direction for infection control and the development of new medication materials in RCT.

## Materials and methods

### Materials

OCT was obtained from Yuanye Bio-Technology. Calcium oxide, PEG, ε-caprolactone (CL) and stannous octoate were obtained from Shanghai Aladdin Bio-Chem Technology Co., Ltd. Trifluoroethanol (TFE) and dimethyl sulphoxide were purchased from Concord Co., Ltd. 1,4,8-trioxa[4.6]spiro-9-undecanone (TOSUO) was prepared in our laboratory according to a published procedure [22]. PECT was prepared from PEG, CL and TOSUO through ring-opening copolymerization as reported previously [23]. CHX hydrogel (2%) and sodium hypochlorite (NaOCl, 5.25%) were obtained from Biotechnology Longly. Ca(OH)_2_ paste was obtained from Ivoclar Vivadent. *Enterococcus faecalis* (ATCC 29212), *S. mutans* (ATCC 700610) and *A. viscous* (ATCC 27044) were purchased from the Guangdong Microbial Culture Collection Center of China.

### Preparation of ALK dual drug-loaded PECT hydrogel

The loading concentration of OCT was determined according to the previous literature [24, 25]. OCT-HCl and OCT were loaded into PECT hydrogel in two steps. First, OCT was loaded into PECT NPs through nanoprecipitation. OCT (20 mg) and PECT (250 mg) were dissolved in TFE (5 ml), which was then gradually added dropwise to double-distilled water (100 ml). Subsequently, the mixture was further stirred at room temperature for 12 h to remove TFE and obtain PECT@OCT NPs. Next, PECT@OCT NPs were freeze-dried into a white powder. To prepare dual OCT-loaded PECT hydrogel (OCT/PECT@OCT), PECT@OCT NPs powder was dissolved in phosphate buffer solution (PBS), and then OCT-HCl was added into the mixture. After magnetic stirring for 30 min to ensure even dispersion, OCT/PECT@OCT hydrogel was obtained.

**Figure 1.**
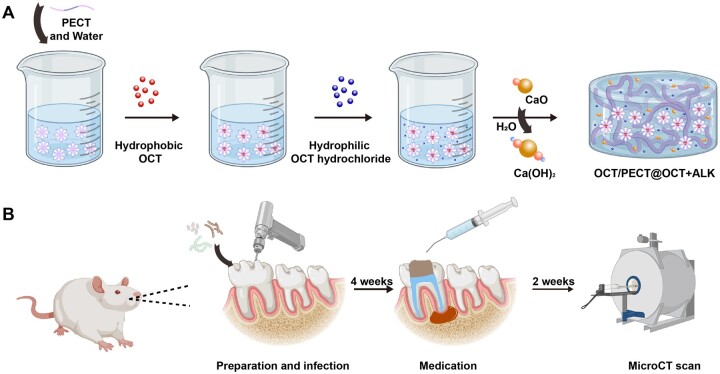
Schematic diagram. (**A**) The preparation of OCT/PECT@OCT + ALK hydrogel. (**B**) The establishment of rat apical periodontitis model and experimental procedure.

To prepare ALK OCT/PECT@OCT hydrogel, 1 mM CaO powder was added into OCT/PECT@OCT hydrogel and stirred in an airtight container to obtain the OCT/PECT@OCT + ALK hydrogel. In this process, CaO can react with H_2_O in NP solution to form Ca(OH)_2_, which eventually dispersed throughout the hydrogel system (Figure 1A).

### Characterizations of the hydrogel

After the preparation of OCT/PECT@OCT + ALK, the size of blank PECT NPs and OCT/PECT@OCT + ALK NPs were measured by dynamic light scattering (DLS) to determine the load of OCT. The sol–gel transition behaviour of OCT/PECT@OCT + ALK aqueous solution was tested using the vial-flip method. The OCT/PECT@OCT + ALK aqueous solution was added into the vial and incubated at 37°C to observe the fluidity in real time. To examine the micromorphology of all types of hydrogels, hydrogel samples were flash-frozen in liquid nitrogen and lyophilized for 72 h. The freeze-dried hydrogel was cut off to acquire cross-sections, and then the sections were sputter coated with gold–palladium in a vacuum evaporator, followed by observation under a scanning electron microscopy (SEM) system (Gemini 300 Zeiss, Germany) at 10.00 kV. Rheological measurements of aqueous OCT/PECT@OCT and OCT/PECT@OCT + ALK aqueous solution were performed under oscillatory and steady shear conditions with a fluid rheometer (MCR 302; Anton Paar, Graz, Austria) set to an automatic gap. OCT/PECT@OCT and OCT/PECT@OCT + ALK aqueous solution were placed between parallel plates (diameter = 10 mm) separated by 1.0 mm. The temperature-sweep test was used to monitor the variation in storage modulus (G′) and loss modulus (G″) at a fixed frequency of 0.1 Hz from 20°C to 60°C at a heating rate of 2°C min^−1^. To further evaluate the injectability and clearance of the hydrogel, Ca(OH)_2_ paste and OCT/PECT@OCT + ALK aqueous solution were separately injected to root-canal models *in vitro* for 7 days to simulate the process of using intracanal medicaments in clinics. After 7 days, the samples were gently irrigated for 1 min with sterile saline (5 ml) to remove intracanal medicaments. All samples were observed under a stereoscopic microscope (Leica S9i, Germany) and SEM.

### Release and degradability of drug and the formation of alkaline environment *in vitro*

The drug release and gel degradation of hydrogels were tested *in vitro*. Each hydrogel sample was placed in tubes and incubated at 37°C for 12 h to form stable hydrogels. These hydrogels were then added fresh PBS (4 ml) at 37°C in a constant-temperature oscillator (100 rpm). The supernatant solution (3 ml) was collected at predefined time intervals and replaced with an equivalent volume of fresh PBS. Three parallel samples were collected from each group. The absorbance of OCT in the supernatant was evaluated by UV spectrophotometry (Beckman Coulter, Brea, CA, USA) and further calculated the real-time concentration of OCT, which released from the hydrogel system. And the accumulated drug release was calculated according to the following formula:

$$E=\left(\frac{V_{E}\sum_{1}^{n-1}C_{i}\;+\;V_{0}C_{n}}{m_{0}}\right)\;\times \;100\%$$

***E*** is the cumulative release; ***V_E_*** is the sampling volume; **V_0_** is the initial release volume; ***C_i_***and ***C_n_*** is the drug concentration; ***i*** and ***n*** are the number of samples; **m_0_** is initial mass of drug in the gel.

Simultaneously, the size of NPs in the supernatant was measured by DLS, and the pH was also monitored. Additionally, the hydrogel was immersed in PBS at 37°C for various time intervals. Degradation was calculated as relative weight by measuring the ratio of the residual weight to the initial weight of the hydrogel, and a degradation curve was constructed.

### Preparation of root-canal models

Freshly extracted human single-rooted teeth, excluding tooth with root caries, cracks and previous endodontic treatment, were collected with informed consent under a protocol approved by the Medical Ethics Committee, Hospital of Stomatology, Tianjin Medical University, China (TMUhMEC20220809). The teeth were debrided of bone, calculus and soft tissue and then stored in 0.9% saline during all procedures to avoid dehydration. A low-speed diamond disc mounted on a milling machine under water cooling was used to section the teeth between cementoenamel junction and the apical third of the root to obtain 6 mm root. All root canals were enlarged to a size of 25 06# (M3 nickel-titanium instrument, Yirui Medical Equipment, China) and rinsed with 0.9% sterile saline and 5.25% NaOCl during preparation. Each single root was prepared with two parallel shallow grooves along the long axis of the tooth with a diamond disc to obtain a sample. After removing the smear layers from the canal walls in an ultrasonic bath with 17% EDTA and 5.25% NaOCl solutions for 5 min per samples, all blocks were autoclaved at 121°C for 30 min in brain-heart infusion broth (BHI) (5 ml). They were finally cultured at 37°C and 5% CO_2_ for 24 h to confirm sterility.

### Multispecies biofilm formation

*Enterococcus faecalis*, *A. viscous* and *S. mutans* are the typical bacterial species of primary and/or secondary endodontic infections were selected [26]. To facilitate bacterial penetration into the dentine tubules, a centrifuge was used with parameters set at 1400*g*, 2000*g*, 3600*g* and 5600*g* for two cycles of 5 min each [27, 28]. A total of 120 sterile root-canal models were randomly placed in 10 ml Eppendorf tubes containing 2 ml of each type of bacteria to combine into a 6 ml suspension. The growth medium for the biofilms comprised BHI medium supplemented with 1% sucrose [29]. The inoculation of the multispecies biofilm occurred in two phases. *Actinomyces viscous* constituted the starting culture, and then *E. faecalis* and *S. mutans* was added after 14 days. The timing and inoculum size for the addition of *E. faecalis* and *S. mutans* was based on preliminary experiments showing these conditions to result in optimal species balance without biofilm overgrowth by *E. faecalis* and *S. mutans*. All Eppendorf tubes were incubated for 21 days at 37°C in 5% CO_2_ for biofilm formation. The medium was changed every second day. After 21 days of incubation, three dentine blocks were randomly selected and subsequently washed with 0.9% sterile saline for 1 min to remove planktonic bacteria. Subsequently, the samples were fixed with 2.5% glutaraldehyde for 2 h in 4°C, washed with ultrapure water, dehydrated in a series of ethanol solutions, dried under a vacuum, sputter coated with gold and finally observed on SEM at 2.0 and 5.0 keV to confirm the formation of multispecies biofilm.

### Intracanal medicament placement

The antimicrobial activity of the intracanal medicaments was tested in the root canals over a 7-day period. To prevent dehydration during the experiment, the outer surface of the root canal blocks was covered with nail varnish. All blocks were stabilized in a vertical orientation with the root-canal opening at the top and embedded firmly into dental wax in the wells of sterile 24-well plates in a random order as mentioned in the previous literature [30]. The inoculated root blocks were randomly assigned to seven groups according to intracanal medicaments: (i) control (0.9% sterile saline) (*n* = 10); (ii) blank PECT hydrogel (*n* = 10); (iii) OCT/PECT@OCT hydrogel (*n* = 20); (iv) OCT/PECT@OCT + ALK hydrogel (*n* = 20); (v) PECT + ALK hydrogel (*n* = 20); (iv) Ca(OH)_2_ paste(*n* = 20); and (vii) 2% CHX hydrogel (*n* = 20). All groups were further divided into two groups based on the length of time of application (3 and 7 days). All groups were further divided into two groups based on the length of time of application (3 and 7 days). All medicaments were delivered once into the root canals, which were then closed with hydraulic temporary restorative (GC Caviton, Japan) to prevent evaporation. All blocks were stored at 37°C in 5% CO_2_ atmosphere and a humid environment.

### Antibacterial activity on liquid medium

To investigate the antimicrobial effect of the hydrogel and correspond to its drug-release profile, daily extract of the gel was obtained and its antiplankton effect was tested. The following hydrogels were placed in the upper chamber of 24-tranwell plates: PECT, PECT + ALK, OCT/PECT@OCT, OCT/PECT@OCT + ALK and CHX gel. PBS solution was added to the lower well plates. The ratio of gel to PBS solution was maintained at 1:4. The gel extract was collected, and the PBS solution was refreshed daily over a period of 7 days. The optical density of the *A. viscous*, *E. faecalis* and *S. mutans* planktonic cultures were adjusted spectrophotometrically to a density of 1 × 10^8^ colony-forming units (CFU)/ml. The gel extract was mixed with each bacterial solution and incubated in a 37°C incubator for 24 h. After incubation, the mixture was diluted to the appropriate concentration, and the diluted bacterial suspension (100 μl) was evenly spread on a BHI agar plate. CFU were counted after a 24 h interval, and bacterial-clearance rate was determined by comparing it with the PBS blank group. The experiment was repeated three times and analysed statistically.

### Evaluation of antibiofilm effect and bacterial viability by SEM and confocal laser scanning microscopy

After 7 days of medication, the samples were gently irrigated with sterile saline to remove intracanal medicaments and prepared to observe the dentine canals on a cross-sectioned surface.

Half of the samples were preprocessed as described above and finally mounted onto the SEM system operated at 2.0 and 5.0 keV. The dentine surface of root canals was scanned at three different random locations and then analysed with Image J software to evaluate the biofilm clearance of different medicaments.

To assess the bacterial viability in biofilm, the other half of the specimens were prepared for confocal laser scanning microscopic (LSM-800, Zeiss) analysis. A fluorescent LIVE/DEAD™ BacLight™ viability kit containing SYTO-9 and propidium iodide (PI) dyes (1:1) was used to stain live and dead bacteria at 4°C in darkness for 15 min. The NO1 probe (green; excitation at 488 nm and emission at 525 nm) was used to label viable bacteria, whereas the PI probe (red; excitation at 540 nm and emission at 620 nm) was used to label dead bacteria. Each sample was scanned at three different locations. The area fraction of viable and dead bacteria was quantitatively analysed, and the proportion of dead bacteria was measured and calculated using Image J.

### *In vivo* evaluation of antibacterial activity

Eight-week-old Sprague-Dawley male rats (200–250 g) were used to establish a root-canal infection model. The four experimental groups used were as follows: OCT/PECT@OCT + ALK gel, Ca(OH)_2_ paste, 2% CHX gel and control group (*n* = 8). All procedures involving animals were approved by the Tianjin University of Traditional Chinese Medicine Ethics Committee (ethics number TCM-LAEC2023109). Animals were anaesthetized by intraperitoneal injection of phenobarbital sodium (40 mg/kg). The distal middle root canals of both sides of the maxillary first molar teeth were chosen as the target sites to establish the model. A #1/4 carbide round bur attached to a high-speed handpiece was used to penetrate and remove the roof of the pulp chamber. The root canals were then manually enlarged to #10 using endodontic k-file preparation. Additionally, the contralateral occlusion was reduced by 1 mm. Subsequently, a total of 15 μl of a polymicrobial mixture containing *E. faecalis*, *S. mutans* and *A. viscous* was injected into the root canals by using a syringe (1 ml) followed by temporarily sealing with glass–ionomer cement for 4 weeks to induce periapical lesion formation. In the fourth week, one rat from each group was sacrificed to evaluate the presence of apical inflammation by micro-CT (Bruker, sky scan 1276, Germany). The remaining rats were anaesthetized, and the root canals were enlarged by using ProtaperM2 nickel–titanium endodontic files up to size 25#04. Alternating rinses with 1% NaClO and 17% EDTA solutions were performed for 1 min, followed by a final rinse with saline by using rinsing needles to simulate standard clinical rinsing procedures. Subsequently, a K-stainless steel hand file up to size #6 was used to assist each group of intracanal medicaments to reach the root canal, and it was temporary sealed with 3M flowable resin [31]. At the end of a 2-week medication, the remaining 28 rats were sacrificed, and only the samples which temporary materials sealed well were selected. And to exclude the effect of sealing materials on CT imaging, the temporary sealing materials were removed and the samples were gently irrigated with sterile saline for 1 min to remove intracanal medicaments for further CT evaluation. The teeth along with both maxillae were removed fixed in 4% paraformaldehyde for 48 h before scanning with micro-CT to evaluate apical bone healing for each group (Figure 1B).

Major organs (heart, kidney, spleen, liver and lung) of rats were collected and immersed in 4% paraformaldehyde, dehydrated, embedded in paraffin and serially cut into 6-μm sections. The sections were stained with haematoxylin and eosin (H&E).

### Statistical analysis

Statistical analysis was carried out by SPSS 25.0. All quantitative data were expressed as the mean ± standard deviation (SD). Statistical analysis of the results was performed using one-way ANOVA and the least significant difference test. A value of *P *<* *0.05 was considered to be statistically significant. Differences were considered indicative of statistical significance at * *P *<* *0.05, ** *P *<* *0.01 and *** *P *<* *0.001, ns means no significance.

## Results

### Preparation and characterization of OCT/PECT@OCT + ALK hydrogel

**Figure 2.**
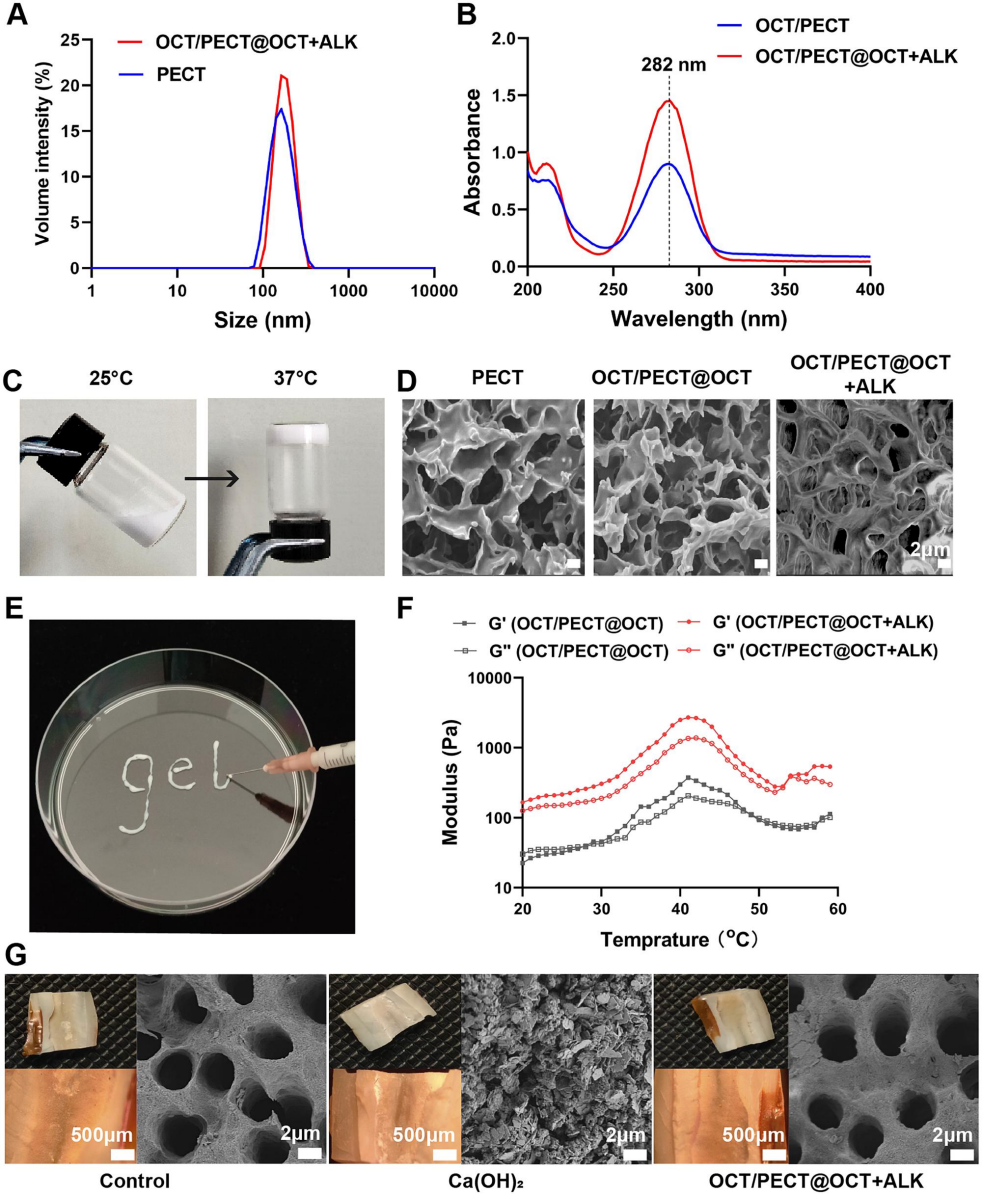
(**A**) Particle size distribution of PECT and OCT/PECT@OCT + ALK nanoparticles. (**B**) The absorbance of OCT encapsulated in the hydrogel. (**C**) Images of the nanoparticle dispersion sol (25°C) and hydrogel (37°C) states of OCT/PECT@OCT + ALK NP aqueous dispersions. (**D**) SEM images of lyophilized PECT hydrogel, OCT/PECT@OCT NP hydrogel and OCT/PECT@OCT + ALK NP hydrogel. (**E**) Injectability of OCT/PECT@OCT + ALK gel. (**F**) Viscosity of OCT/PECT@OCT and OCT/PECT@OCT + ALK NP aqueous dispersions as a function of temperature at 25% (w/w). (**G**) Images of root canal surface after removing intracanal medicaments: camera without magnification, stereoscopic microscope magnification 20× and SEM image × 5.0 keV.

Owing to the amphiphilic property of PECT polymer, it can self-assemble into NPs with a regular hydrophilic shell around a hydrophobic core, whereas the hydrophobic drug can be loaded into the hydrophobic core in a stable form. Then, the PECT NP solution were connected by inter-micellar bridges, resulting in higher sol-to-gel transition temperature. To meet the need of local antimicrobial dose in the treatment of apical inflammation, the hydrophilic OCT was encapsulated in the hydrophilic part of hydrogel system to instantaneously elevate the local drug concentration through burst release. The hydrophobic OCT was further loaded into the PECT NPs to achieve a slower and sustained-release profile. As shown in Figure 2A, PECT and OCT/PECT@OCT + ALK NPs showed similar particle sizes about 162 nm and a narrow distribution, confirming that the hydrophobic OCT, was encapsulated into the PECT NPs. Additionally, the maximum absorbance of hydrophilic and hydrophobic OCT encapsulated in the hydrogel was maintained at 282 nm (Figure 2B). And to enable a local alkaline (ALK) condition, low-concentration Ca(OH)_2_ was further dispersed in the hydrogel system to obtain the final OCT/PECT@OCT + ALK hydrogel. Vial-flip test demonstrated that OCT/PECT@OCT + ALK aqueous solution was a milky white solution at room temperature (25°C) and transformed into hydrogel at 37°C in a short time, as shown in Figure 2C. The internal morphology was characterized, and SEM images revealed a uniform, continuous porous structure in all three hydrogels (Figure 2D). These pores microstructure enabled the flow of media and transportation of drugs within the hydrogel matrix. Additionally, SEM images of OCT/PECT@OCT + ALK gel exhibited a uniform and more compact porous structure without obvious Ca(OH)_2_ precipitation, suggesting that the OCT/PECT@OCT + ALK gel was a uniformly dispersed system.

To elucidate the thermosensitive property of OCT/PECT@OCT + ALK gel, we measured the variations in storage modulus (G′) and loss modulus (G″) of OCT/PECT@OCT and OCT/PECT@OCT + ALK hydrogel from 20°C to 60°C through rheological analysis. As depicted in Figure 2F, OCT/PECT@OCT gel and OCT/PECT@OCT + ALK gel exhibited typical thermosensitive properties. With increased temperature, the gel modulus increased and the maximum storage modulus was observed at 35–45°C due to the convergence and aggregation of NPs induced by higher temperature. However, with further increased temperature, the modulus decreased, indicating the disruption of the hydrogel crosslinking network. Notably, compared with OCT/PECT@OCT gel, OCT/PECT@OCT + ALK gel exhibited higher modulus, but the OCT/PECT@OCT + ALK gel still showed good injectability at room temperature (Figure 2E). The images of the root-canal surface after irrigation showed that few residues on the dentine surface under the camera and stereoscopic microscope in the Ca(OH)_2_ group, whereas a large accumulation of residual Ca(OH)_2_ covered the dentine surface, as observed under SEM. The OCT/PECT@OCT + ALK gel group exhibited good root-canal clearance under microscopic observations and SEM (Figure 2G).

### Drug release and degradability of the hydrogel *in vitro*

**Figure 3.**
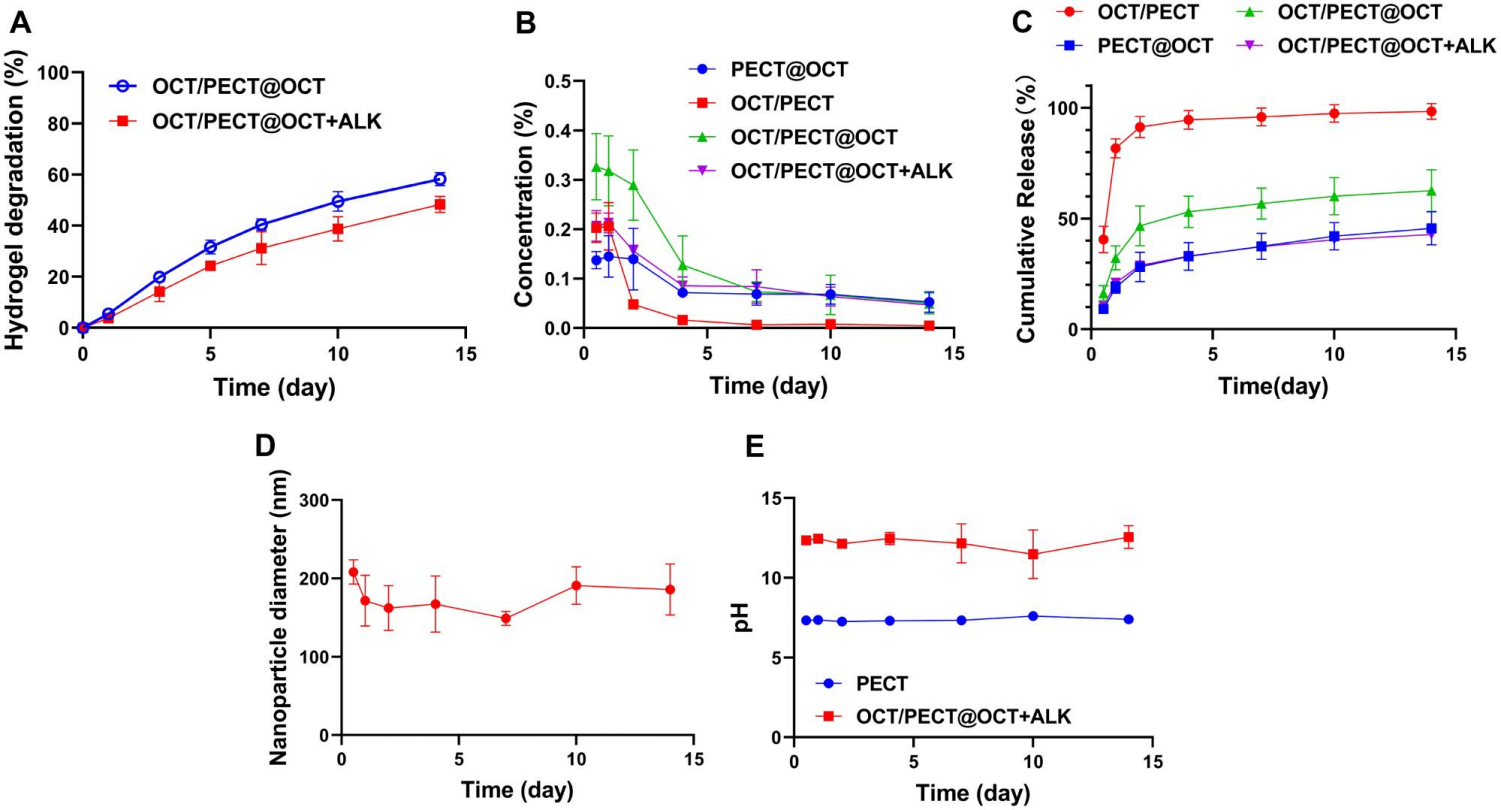
(**A**) Degradation curve of OCT/PECT@OCT and OCT/PECT@OCT + ALK hydrogels. (**B**) The real-time concentration of OCT release profiles at 37°C from the hydrogels formulation. (**C**) The cumulative release profiles of the hydrogels formulation. (**D**) The temporal change of OCT/PECT@OCT + ALK hydrogel nanoparticle diameter over a 14-day period. (**E**) The pH of hydrogel supernatant.

The degradability of the OCT/PECT@OCT + ALK hydrogels was further determined *in vitro*. As shown in Figure 3A, OCT/PECT@OCT + ALK and OCT/PECT@OCT hydrogels displayed continuous degradation during the observation period. On the 14th day, the degradation rates were found to be 58.2% and 48.3% for the two hydrogels, respectively. Notably, the addition of Ca(OH)_2_ to OCT/PECT@OCT + ALK hydrogel resulted in a lower degradation rate than that of OCT/PECT@OCT hydrogel, which can be attribute to the higher mechanical modulus. As shown in Figure 3B and C, the hydrophilic OCT exhibited faster drug release, in which the drug concentration of OCT reached the maximum in the initial 2 days followed by a rapid decline and the cumulative release of OCT/PECT hydrogel almost reached to 100%. The reason may be the dependence of the release of the hydrophilic drug in the hydrogel on physical diffusion [32]. Conversely, the hydrophobic OCT encapsulated in the PECT hydrogel showed a more sustainable release profile, in which the drug-release concentration can maintain 1 mg/ml for 14 days and the cumulative release curve of PECT@OCT hydrogel was relatively flat. It was owing to that the hydrophobic drug was released as NPs with the hydrogel degradation, which can prolong the drug-release period and also improve the long-term stability of the loaded drug [33, 34]. After dual drug encapsulation, the OCT/PECT@OCT and OCT/PECT@OCT + ALK hydrogels exhibited high drug concentration at the initial 2 days. It can also maintain effective drug concentration in the following 14 days. The concentration of released OCT during the initial three days was lower than that of gels without Ca(OH)_2_ owing to the more compact microstructure of the OCT/PECT@OCT + ALK hydrogel. After 3 days, both types of gels returned to a steady state as characterized by the long-term slower drug release, maintaining concentrations of around 1 mg/ml. To further certificate the drug release mechanism of OCT/PECT@OCT + ALK hydrogel, the release medium was monitored by DLS. As shown in Figure 3D, NPs with an average diameter of 175 nm were observed in the release medium during the whole releasing process of OCT/PECT@OCT + ALK hydrogel, confirming that the release mechanism for hydrophobic OCT was based on OCT-loaded PECT NPs.

The pH changed within a 14-day period after the addition of 1M CaO into the hydrogel system is illustrated in Figure 3E. The degradation medium from blank PECT exhibited a neutral solution with a pH of around 7.2. Upon the addition of CaO, the degradation medium displayed a significantly elevated ALK pH of around 12.4, which was maintained throughout the entire monitoring period.

### *In vitro* evaluation of antibacterial activity

**Figure 4.**
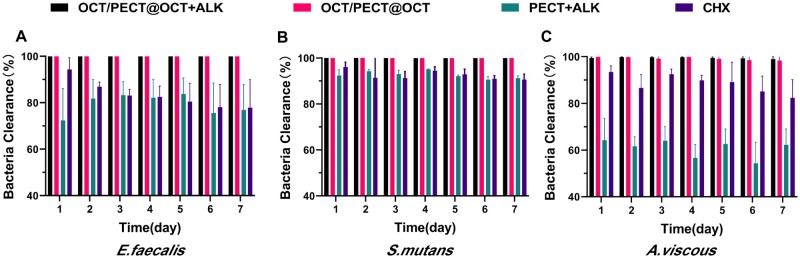
Clearance ratio of plankton bacteria of *E. faecalis* (**A**), *S. mutans* (**B**) and *A. viscous* (**C**).

As shown in Figure 4, during the 7-day antibacterial test, OCT/PECT@OCT + ALK and OCT/PECT@OCT hydrogel extracts exhibited bactericidal rates exceeding 98% against *E. faecalis*, *S. mutans* and *A. viscous* bacterial liquid and the two columns were basically equal. PECT + ALK and CHX hydrogel extracts had similar bactericidal rates against *E. faecalis* (above 60%) and *S. mutans* (above 85%), whereas the bactericidal rate of CHX (75–95%) was higher than that of PECT + ALK (50–75%) against *A. viscous.*

**Figure 5.**
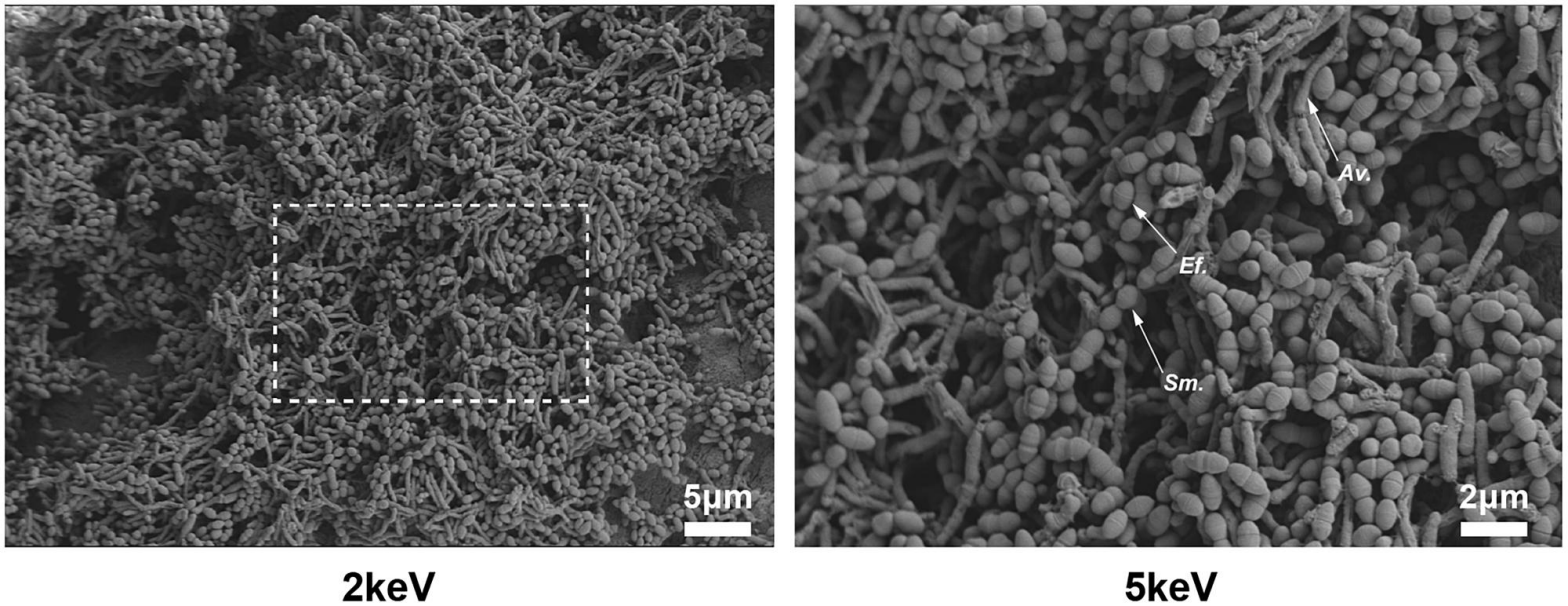
SEM images of multispecies biofilms on dentine blocks magnification: ×2keV and ×5keV, indicated by arrows. *Ef*.: *E. faecalis*; *Sm*.: *S. mutans*; *Av*.: *A. viscous*.

SEM images revealed that after 21 days of cultivation, the dentine surface of the root-canal model was completely covered with a dense and mature multispecies biofilm comprising *E. faecalis*, *S. mutans* and *A. viscous* bacteria, which adhered onto one another and stacked (Figure 5).

**Figure 6.**
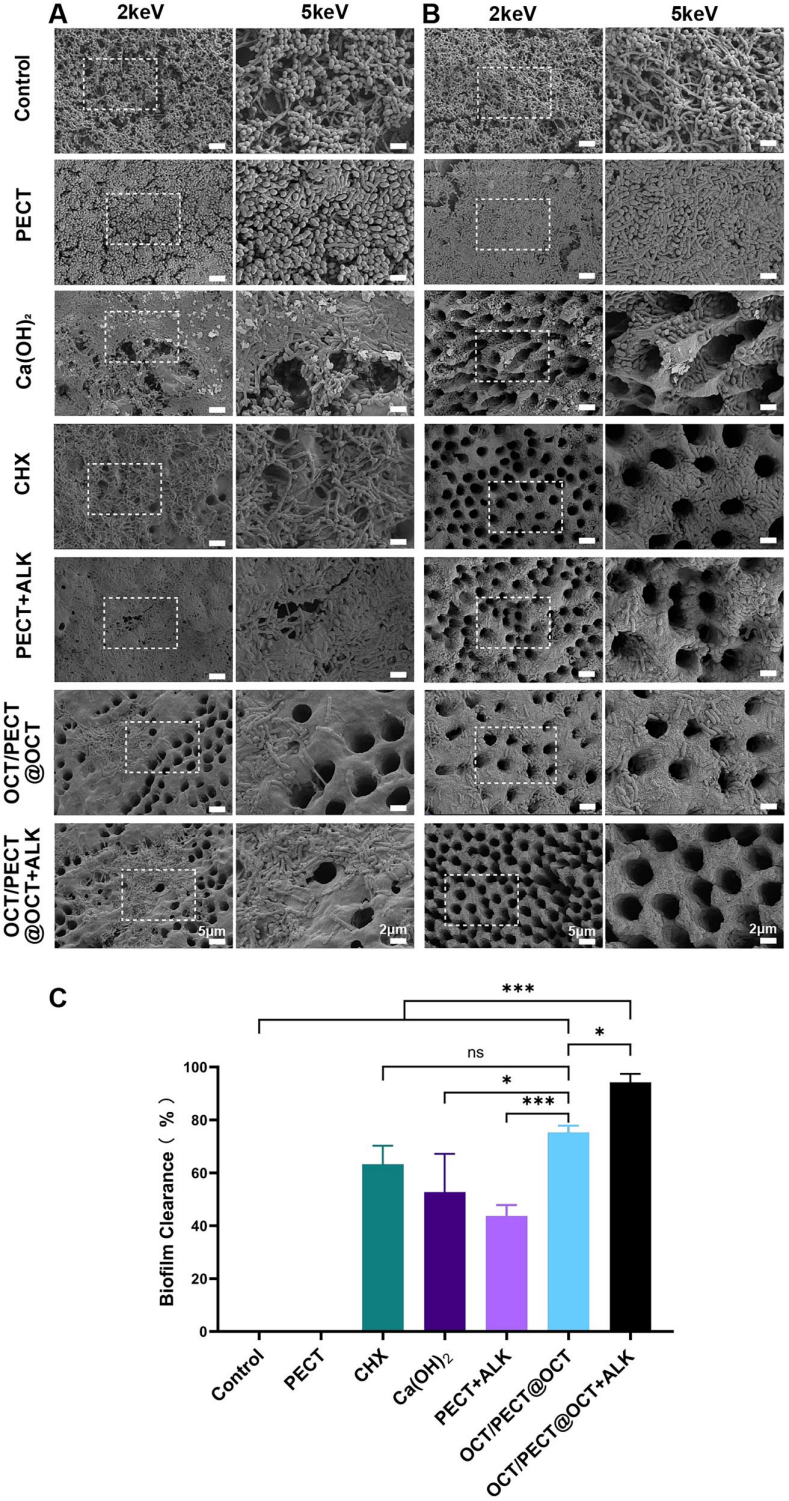
SEM images of multispecies biofilms of the root canal surface: (**A**) after 3 days medication, (**B**) after 7 days medication. (**C**) The biofilm clearance ratio after 7 days medication under SEM. Statistical significance is indicated as *P <* *0.05, **P <* *0.01, ***P <* *0.001, ns means no significance.

Day 3 was selected as the intermediate sampling time based on the previously determined drug-release curve. After 3 days of medication, SEM images revealed that the biofilm of the CHX, Ca(OH)_2_ and PECT + ALK groups had small defects, whereas most biofilm was cleared in the OCT/PECT@OCT and OCT/PECT@OCT + ALK groups. The remaining bacteria showed different extents of cell membrane damage (Figure 6A), and the underlying dentine tubule surface was exposed.

At the end of a 7-day medication, the biofilm area decreased in all experimental groups and was effectively eradicated in the OCT/PECT@OCT + ALK group (Figure 6B). As analysed in Figure 6C, the biofilm clearance ratio of OCT/PECT@OCT + ALK group (94.21% ± 3.23% (SD)) was significantly the highest among all groups, followed by OCT/PECT@OCT (78.54% ± 7.30% (SD)) (*P *<* *0.001 and compared with the OCT/PECT@OCT gel group (*P < *0.05)).

**Figure 7.**
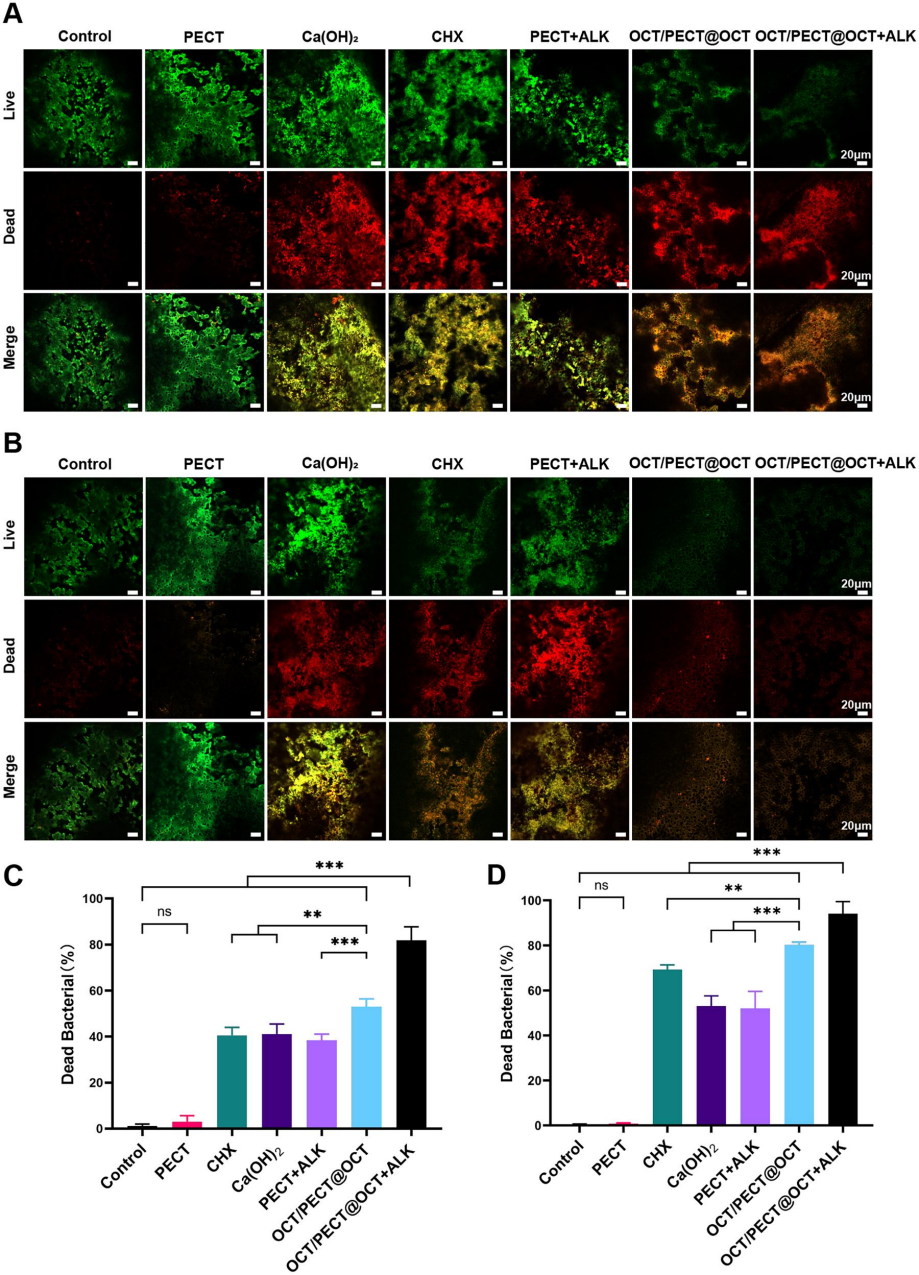
Live/dead bacteria in stained biofilm of the root canal surface: (**A**) after 3 days medication, (**B**) after 7 days medication (scale bars = 20 μm.). The proportion of dead bacteria in biofilms under CLSM: (**C**) after 3 days medication, (**D**) after 7 days medication. Statistical significance is indicated as *P <* *0.05, **P <* *0.01, ***P <* *0.001, ns means no significance.

The distribution of viable and dead bacteria on dentine surfaces was observed via Live/Dead staining to further evaluate the antimicrobial efficacy of medication. After 3 days, substantial live bacteria (green) remained in Ca(OH)_2_ paste, CHX and PECT + ALK hydrogel groups. However, OCT/PECT@OCT and OCT/PECT@OCT + ALK gel groups were dominated by dead bacteria (red) (Figure 7A). The proportion of dead bacteria of OCT/PECT@OCT + ALK group (81.87% ± 5.92% (SD)) was the highest among all groups (*P *<* *0.001) (Figure 7C). After 7 days, the proportion of dead bacteria in other groups increased, and only a minimal amount of red fluorescence can be observed in OCT/PECT@OCT and OCT/PECT@OCT + ALK (Figure 7B). The proportion of dead bacteria of OCT/PECT@OCT + ALK was still the highest (98.01% ± 1.10% (SD)) (*P *<* *0.001), followed by OCT/PECT@OCT (89.47% ± 1.51% (SD)) (Figure 7D). There was no significant difference between PECT hydrogel and control group, which meant that PECT hydrogel had negligible antibacterial effect.

### *In vivo* evaluation of antibiofilm activity

**Figure 8.**
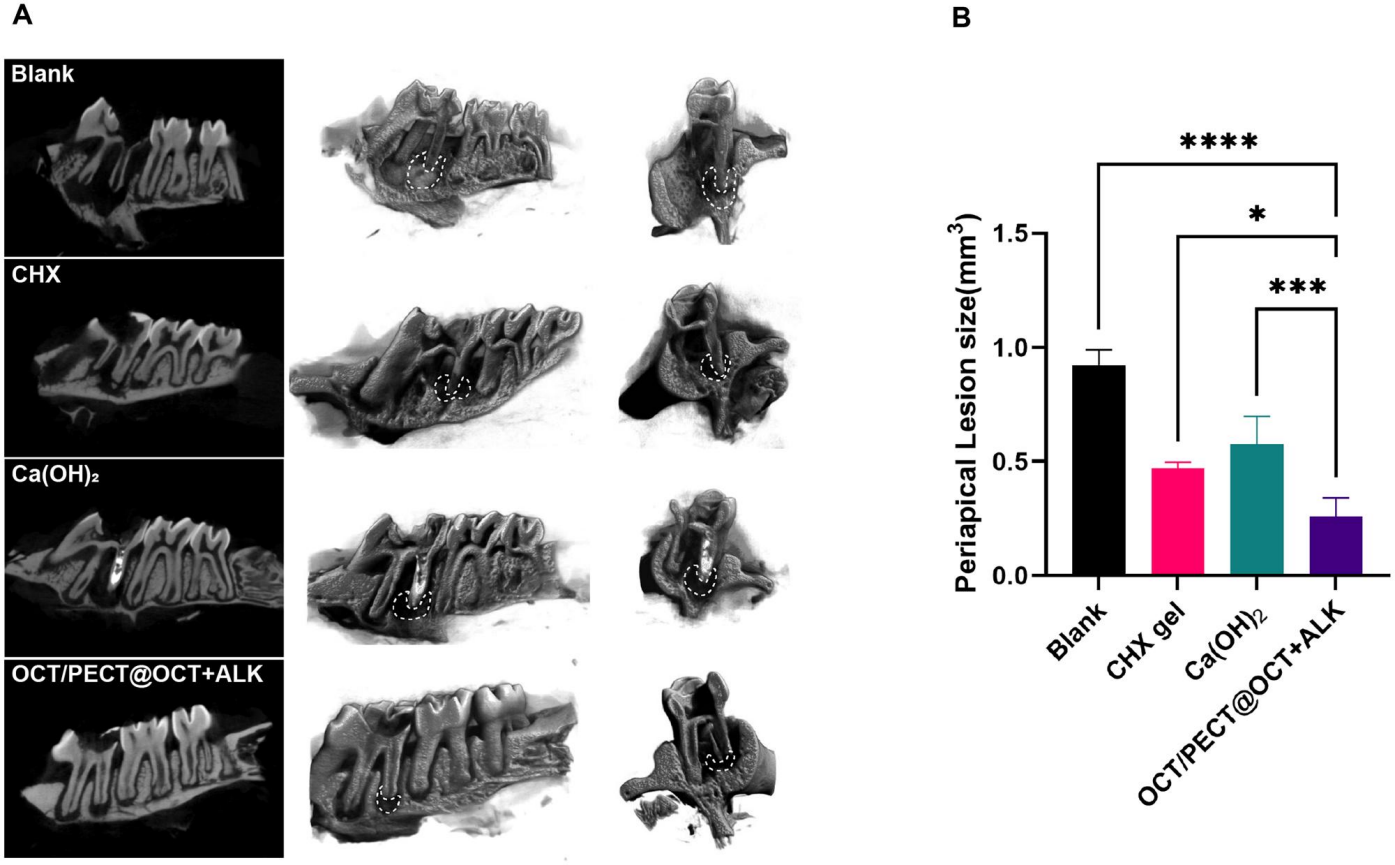
Evaluation of periapical inflammation after using intracanal medicaments of infected root canals *in vivo*. (**A**) Micro-CT and 3D reconstruction images of periapical inflammation in blank, CHX gel, Ca(OH)_2_ paste and OCT/PECT@OCT + ALK gel groups. (**B**) Image of periapical lesion size (mm^3^). Statistic were analysed by CTvox and SPSS 25.0. Statistical significance is indicated as *P <* *0.05, **P <* *0.01, ***P <* *0.001, ****P <* *0.0001.

A period of 2-week medication of four intracanal medicaments was conducted after the successful establishment of the rat periapical inflammation model. After 2 weeks, all rats were sacrificed. Their teeth along with bilateral maxillae were removed for micro-CT analysis and 3D reconstruction by using CTvox (Bruker, sky scan 1276, Germany) to examine the healing of periapical inflammation (Figure 8A). As analysed in Figure 8B, the periapical lesion size of OCT/PECT@OCT + ALK (0.26 ± 0.07 (SD) mm^3^) was significantly the smallest among all groups (*P *<* *0.001 and compared with the CHX gel group (*P *<* *0.05)).

**Figure 9.**
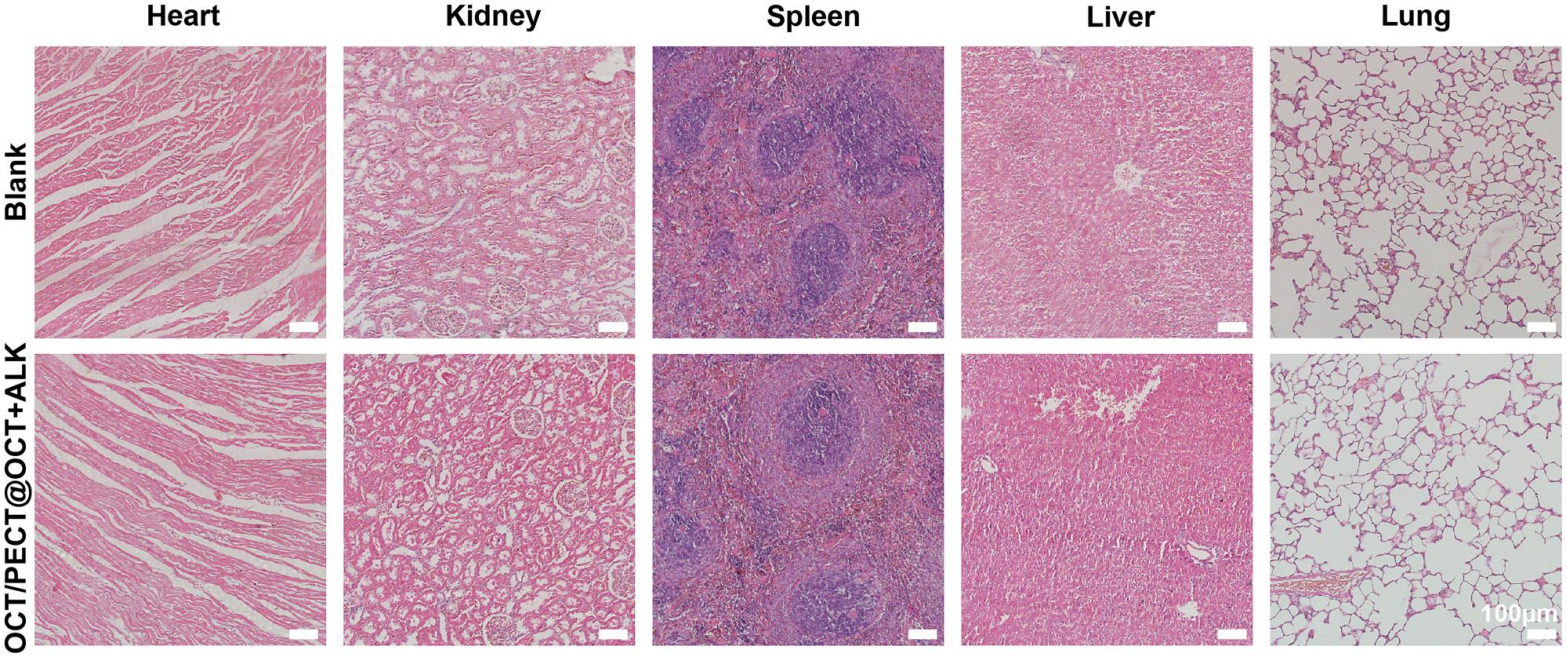
H&E-stained images of major organs (heart, kidney, spleen, liver and lung) obtained from the OCT/PECT@OCT + ALK gel group and the blank group (scale bars = 100 μm).

H&E staining revealed that the OCT/PECT@OCT + ALK hydrogel did not damage the pathological structure of rats main organs, guaranteeing the biosafety of the hydrogel *in vivo* (Figure 9).

## Discussion

Exploring intracanal medicaments with efficient and long-term antimicrobial effects is an essential direction of current research. In this study, PECT hydrogel was used to load with hydrophilic and hydrophobic OCT and incorporated with Ca(OH)_2_, which had the dual antimicrobial effects of sustained drug release and local ALK condition. The persistent antimicrobial effect was combined with the convenience of clinical operation, which provided an option for using intracanal medicaments and replacing the current disinfectant like Ca(OH)_2_ paste after root-canal preparation.

Hydrogels, the current preferred material for topical medications, can be classified as conventional and smart hydrogels [35]. Among them, the formation of smart hydrogels was primarily related to environmental changes (pH, temperature, pressure, special wavelength light, etc.) [36]. As a relatively closed space, the tooth root canal exchanges substances with the body only through the apical foramen with little environmental changes. Therefore, alterations in body temperature enable thermosensitive hydrogels to be a more advantageous alternative in the selection of intracanal medicaments. Hydrogels exhibit a liquid state at room temperature, demonstrating excellent injectability to accommodate the intricate morphology of the root canal. It rapidly transforms into a gel upon injection into the root canal at body temperature. In the current work, PECT NPs loaded with OCT retained their temperature sensitivity without alteration. With gradually increased temperature, PECT NPs aggregated to form a gel-mesh structure, and the addition of Ca(OH)_2_ did not affect this process. However, it was dispersed in the hydrogel mesh structure, thereby improving the hydrogel strength. Rheological tests showed that the curve of G′ and G″ in OCT/PECT@OCT hydrogel intersected at ∼28°C. However, its maximum storage modulus was located at 35–45°C, indicating enhanced stability at body temperature. Clinical performance was consistent with these findings; nevertheless, long-term preservation is still recommended at low temperatures due to environmental instability.

Our experimental results further demonstrated a significant increase in the modulus of the composite hydrogel following the addition of Ca(OH)_2_. Notably, the G′ and G″ curves did not intersect. The absence of an intersection can be attributed to the rheological test, which involved a warming process ranging from 20°C to 60°C. During this process, the gel was exposed to air, allowing Ca(OH)_2_ to react with CO_2_ in the air to generate calcium carbonate (CaCO_3_) precipitate, so it was already in the gel state at room temperature. However, at this time, the gel modulus was small and still had good injectability. The gel modulus increased with increased temperature, forming a more stable and robust hydrogel. Previous studies have indicated that Ca(OH)_2_ stored in different containers underwent slow CaCO_3_ formation within 2 years without significantly affecting its injectability and pH [37]. Therefore, in using this hydrogel, adequate sealing measures can effectively reduce the curing process while ensuring no adverse impact on clinical effectiveness. Moreover, similar to the clinical situation, the Ca(OH)_2_ group showed apparent residues on the surface, resulting in high-density shadows visible in rat experiments. The residual Ca(OH)_2_ within the root canals was challenging to remove entirely, leading to a lengthier clinical operation time, compromised tightness of root-canal filling and even endodontic therapy failure [38]. Conversely, OCT/PECT@OCT + ALK hydrogel with a lower concentration of Ca(OH)_2_ did not form crystals on the root-canal walls after its introduction into the PECT gel system and maintained a relatively stable ALK environment for a longer time. It can be removed by saline rinsing, leaving no apparent residue on the root-canal wall, consistent with the microscopic observation of SEM in the root-canal model *in vitro* and in the animal experiments *in vivo*.

*In situ* thermosensitive hydrogels has been widely used as injectable localized drug depots, which hold the unique characterization of the free-flowing sol state at lower temperatures and transition to the gel state instantly at body temperature, affording a distinctive advantage since they can be implanted by injection instead of surgery [39–41]. One of the examples is Oncogel^®^, a thermosensitive hydrogel of PEG–poly(lactide-co-glycolide acid) (PLGA)–PEG developed by Macromed Company loaded with PTX, which has entered a Phase II clinic trial. And there were many other amphiphilic block copolymers based thermosensitive hydrogel systems that has been widely studied such as PLGA–PEG–PLGA (ReGel^®^), poly(ethylene oxide)–poly(propylene oxide)–poly(ethylene oxide) (PEO–PPO–PEO, Pluronic^®^) and PCL–PEG–PCL and so on. However, ReGel^®^ is a kind of viscous semisolid at ambient temperature with inconveniences in weight and transference. The preparation of drug-loaded PEG–PCL hydrogel generally dissolved at higher temperature and required a pre-quenching treatment. Among them, PECT is a new thermosensitive polymer developed by our lab previously comprising PCL–PEG–PCL amphiphilic copolymers with TOSUO moieties incorporated in the PCL block. The modification reduced the crystallinity of PCL and increased the hydrophilicity of the hydrophobic phase [42, 43]. Therefore, the freeze-dried powders of the drug-loaded PECT NPs could be quickly dispersed in water at room temperature to reconstitute the injectable hydrogel system and form a gel *in situ* after injection *in vivo*, which is convenient for formulation storage and clinical application [44]. Previous study also demonstrated that PECT gels had much greater gel strength and a longer gel lifetime *in vivo* than Pluronic^®^, suggesting a possibly longer drug retention time [45]. More importantly, owing to the amphiphilic property of PECT polymer, it can self-assemble into NPs with a regular hydrophilic shell around a hydrophobic core, enabling the simultaneous loading of hydrophobic and hydrophilic drugs in the hydrogel system to exert diverse functionalities. Therefore, we chose PECT as localized drug depot to realize programmed dual-OCT release. Accordingly, our investigation focused on specializing and refining the programmed release process, which was achieved by simultaneous loading of the hydrophilic OCT in the hydrophilic region and encapsulating the hydrophobic OCT in the hydrophobic region within the core-shell structure of the PECT NPs. Additionally, our study demonstrated that the burst release of the hydrophilic drug at the initial stage of hydrogel application resulted in a rapid eradication of bacteria in the root canal. Conversely, the long-term release of the hydrophobic OCT, synchronized with hydrogel degradation, maintained a consistent concentration of antimicrobial agents in the root canal.

Multispecies biofilm, a common form of root-canal bacteria, provided a more realistic representation of the complexity of root-canal infections [46, 47]. Thus, it was used in our study as a crucial method of assessing the antimicrobial efficacy of intracanal medicaments. Most bacteria remaining in the root-canal wall were *E. faecalis* in Ca(OH)_2_ and PECT + ALK, consistent with a previous proposal that interbacterial and dentine adhesion contribute to bacterial drug resistance, subsequently leading to increased resistance against ALK environments [48]. Conversely, OCT/PECT@OCT and OCT/PECT@OCT + ALK hydrogels demonstrated robust resistance against planktonic bacteria and multispecies biofilm disruption in both short and longer antimicrobial experiments. On the one hand, the loaded OCT, known for its bipyridyl cationic properties, demonstrated the ability to permeate the biofilm matrix, thereby interfering with the formation of bacterial cell membranes and cell walls, leading to rapid sterilization [49]. In previous studies, the antimicrobial properties of OCT were assessed primarily through the antibiofilm effect of *E. faecalis* biofilm [50], so the antimicrobial effect of multispecies biofilm of OCT demonstrated in the study can serve as a valuable theoretical basis for the potential application of OCT in RCT. On the other hand, the programmed drug release of the two hydrogels provided a more efficient biofilm-elimination effect. As shown by the drug-release and degradation curves of the two hydrogels, the increase in Ca(OH)_2_ affected the release of hydrophilic OCT to a certain extent. However, the combination of antibiofilm and animal experimental results confirmed that the increase in ALK environment played a synergistic antibiofilm effect and that the OCT/PECT@OCT + ALK was significantly superior to the OCT/PECT@OCT hydrogel. In addition, we believed that this composite antimicrobial profile may reduce the risk of developing bacterial resistance [51].

Therefore, OCT/PECT@OCT + ALK thermosensitive hydrogel, utilizing the amphiphilicity of PECT hydrogel and the characteristics of controlled-release drugs, offered a dual-mode approach to eliminate bacteria. It featured a short-term burst release and a long-term sustained drug-release profile. This innovation presented a promising approach to more effectively eradicating root canal and periapical infections and preventing reinfections. Meanwhile, the introduction of Ca(OH)_2_ into the hydrogel system retained an antimicrobial effect with its high ALK pH and partially addressed the challenges associated with the cumbersome clinical application of Ca(OH)_2_ paste.

## Conclusion

In summary, we prepared a novel OCT/PECT@OCT + ALK hydrogel as an intracanal medicament for RCT. The innovative hydrogel encapsulated dual hydrophilic and hydrophobic OCT and established a distinctive antimicrobial mode. Through both *in vitro* and *in vivo* experimentation, we confirmed that the hydrogel possessed both short and longer antimicrobial efficacy and provided a local ALK environment in root canals, improving the biofilm clearance rate and the apical inflammation reduction. Furthermore, it had superior properties to common intracanal medicaments, which were easy to operate and irrigate without residues in the clinic and provided a new strategy for the treatment of apical periodontitis.
